# Deep learning-based robust positioning for all-weather autonomous driving

**DOI:** 10.1038/s42256-022-00520-5

**Published:** 2022-09-08

**Authors:** Yasin Almalioglu, Mehmet Turan, Niki Trigoni, Andrew Markham

**Affiliations:** 1 Department of Computer Science, University of Oxford, Oxford, UK; 2 Department of Computer Engineering, Bogazici University, Istanbul, Turkey

**Keywords:** Computer science, Computational science

## Abstract

Interest in autonomous vehicles (AVs) is growing at a rapid pace due to increased convenience, safety benefits and potential environmental gains. Although several leading AV companies predicted that AVs would be on the road by 2020, they are still limited to relatively small-scale trials. The ability to know their precise location on the map is a challenging prerequisite for safe and reliable AVs due to sensor imperfections under adverse environmental and weather conditions, posing a formidable obstacle to their widespread use. Here we propose a deep learning-based self-supervised approach for ego-motion estimation that is a robust and complementary localization solution under inclement weather conditions. The proposed approach is a geometry-aware method that attentively fuses the rich representation capability of visual sensors and the weather-immune features provided by radars using an attention-based learning technique. Our method predicts reliability masks for the sensor measurements, eliminating the deficiencies in the multimodal data. In various experiments we demonstrate the robust all-weather performance and effective cross-domain generalizability under harsh weather conditions such as rain, fog and snow, as well as day and night conditions. Furthermore, we employ a game-theoretic approach to analyse the interpretability of the model predictions, illustrating the independent and uncorrelated failure modes of the multimodal system. We anticipate our work will bring AVs one step closer to safe and reliable all-weather autonomous driving.

## Abstract (web summary)

Changing weather conditions pose a challenge for autonomous vehicles. Almalioglu and colleagues use a geometry-aware learning technique that fuses visual, lidar and radar information, such that the benefits of each can be used under different weather conditions.

## Main

Autonomous vehicles (AVs) have recently attracted considerable attention from academia, industry and the general public due to their potential to revolutionize transportation, accelerated by advances in artificial intelligence. The deployment of AVs in our environmental landscape has the potential to decrease road accidents and traffic congestion, as well as improve our mobility in overcrowded cities. Despite extraordinary efforts from many of the leading names in the AV industry and research, AVs are still out of reach except in limited trial programs due to key concerns on their reliability and safety^1^ (see Supplementary Note 1 for details on AV safety levels). Apart from the technical problems, adverse weather conditions such as rain, fog and snow pose substantial challenges for safe and reliable driverless technology^2,3^.

Autonomous vehicles are equipped with different types of sensors such as cameras, lidars, radars, ultrasound and GPS to achieve a higher level of awareness of the surroundings, leading to increased safety, efficiency and capabilities^2,4^. Along with multiple sensors, artificial intelligence methodologies, machine learning, deep learning and large datasets play major roles in the development of AVs with higher levels of intelligence and mobility^5,6^. Artificial intelligence systems efficiently process the vast amount of multisensory data to train and validate the family of machine learning models that underpin the perception, localization, prediction and motion planning capabilities of autonomous driving systems^7,8^. These systems make sense of the world and the objects in the environment and dictate the paths that the vehicles ultimately take.

The localization capability is responsible for precisely predicting the AV’s position on a map. Most of the core components of AVs such as prediction and planning rely on precise localization to, for example, within a few centimetres. Although AVs heavily rely on signals from space-based global navigation satellite systems such as GPS for localization, radio signals can be lost or degraded in many environments due to obstacles or reflections. In particular, AV operation in urban areas surrounded by high-rise buildings remains highly challenging. In addition, GPS merely provides metre-level location accuracy without orientation information, which is potentially fatal for passengers of AVs or those in the surroundings. For example, an AV might detect itself in the wrong lane before a turn, or might stop too late at an intersection due to imprecise localization. Ego-motion estimation (also called odometry) with onboard sensors provides a complementary localization solution in challenging environments, predicting the accurate relative self-position of AVs. It is therefore an essential component that lies at the core of an autonomous driving algorithmic stack and serves as the basis for numerous algorithms such as localization, prediction and motion planning. A robust and reliable ego-motion estimation system should address the sensor vulnerabilities that might be caused by various factors such as poor environmental conditions and sensor imperfections.

Artificial intelligence in AV research and development relies heavily on the use of public datasets in the computer vision and robotics communities^9^. Although the datasets are ever-increasingly massive, the acquisition of accurate ground-truth data to supervise the artificial intelligence systems is limited due to the need for manual labelling and deficiencies of the existing sensors. Cameras and lidars constitute the two primary perception sensors that are commonly adopted in AV research; however, as these sensors operate in the visible and infrared spectrum, inclement weather dramatically disrupts their sensory data, causing attenuation, multiple scattering and absorption^10^ (Supplementary Note 2). Millimetre-wave radars provide a key advantage over visible spectrum sensors in their immunity to adverse conditions, for example, they are agnostic to scene illumination and airborne obscurants^10,11^. The wavelength of millimetre-wave radars is much larger than the tiny airborne particles that form fog, rain and snow, and hence easily penetrates or diffracts around them. Furthermore, as they are radiofrequency-based sensors, radars do not require optical lenses and can be integrated into plastic housings, making them highly resilient to water and dust ingress. We therefore believe that odometry approaches utilizing millimetre-wave radars will allow robust ego-motion estimation under diverse settings such as day, night, rain, fog and snow, and address the challenges in implementing radars (which are described in Supplementary Note 3). The introduction of a high-resolution radar in AV datasets created new opportunities for ego-motion estimation under challenging conditions. Despite the improved measurements, the radar measurements are still much coarser and noisier than those of lidars and cameras. As a result, ego-motion techniques developed for lidars cause large motion errors. Although further information in the full AV software stack from passive sensors (for example, wheel encoders and inertial measurement units) and intermediate predictions of software modules (for example, loop closure and bundle adjustment) can supplement the ego-motion estimation module, perception sensors such as the camera, lidar and radar play a pivotal role in the performance^12^. Ego-motion estimation methods should therefore exploit the advantages of cameras (rich, dense visual information), lidars (fine granularity within visible range) and radars (immunity to inclement weather) while addressing their relative shortcomings. Although deep learning models offer state-of-the-art solutions for ego-motion estimation tasks (Supplementary Note 4), adverse weather conditions pose a host of substantial challenges such as reduced sensing capability (for example, due to the occlusions caused by precipitation) and a wide range of domain shifts (for example, due to the discrepancy between a training dataset and the data encountered during deployment).

**Fig. 1:**
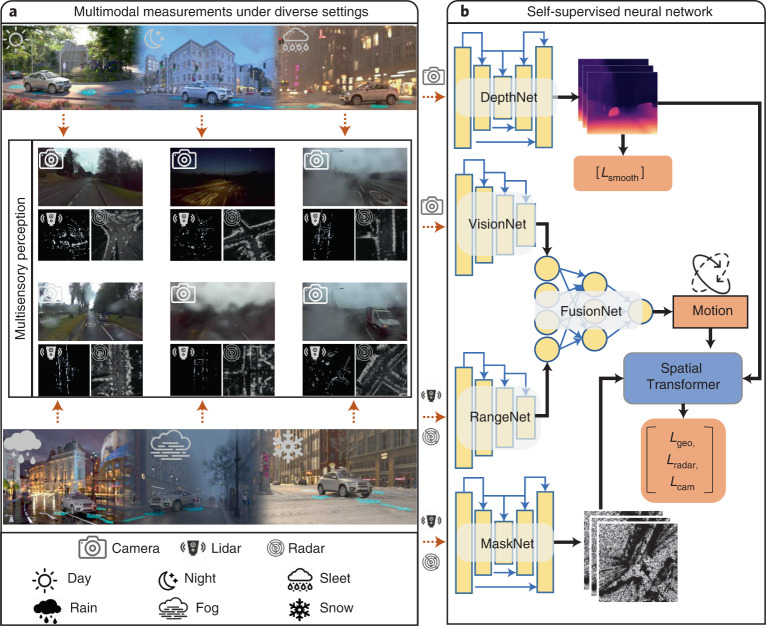
Overview of the GRAMME conceptual framework and architecture. **a**, The publicly available independent AV datasets are collected using multiple sensors such as camera, lidar and radar under diverse settings such as variable ambient illumination and precipitation. Example multimodal measurements from the RADIATE dataset^17^ are shown to illustrate the data types and the degradation in sensor measurements caused by adverse conditions. **b**, Architecture overview for self-supervised estimation of scene geometry and ego-motion. DepthNet and VisionNet modules predict the pixel-wise depth map of each camera frame and the ego-motion between consecutive camera frames, respectively. In parallel, the RangeNet and MaskNet modules operate on range sensors (that is, lidar and radar) to predict ego-motion and input masks, respectively. FusionNet collects the unaligned individual motion predictions as input and predicts the ultimate motion. Finally, the spatial transformer module uses the multimodal predictions and geometrically reconstructs the scene, creating a supervisory signal (L).

Here we propose a novel self-supervised deep learning framework, geometry-aware multimodal ego-motion estimation (GRAMME; Fig. 1) that addresses the key ego-motion estimation challenges for AVs outlined above. Our novel multimodal geometric reconstruction algorithm and reciprocal training technique create a supervisory signal for the self-supervised neural network. Under five diverse settings (day, night, rain, fog and snow) using publicly available independent datasets, we show that our multimodal approach provides robustness to unfavourable weather conditions and outperforms state-of-the-art ego-motion estimation approaches. Following a challenging experimental protocol, we show that the proposed modular design improves the performance of individual modalities even if the other modalities are unavailable at test time, providing robustness to sensor failures. Furthermore, we demonstrate the generalization capability of GRAMME by showing that models trained on regular sequences typically targeted by self-supervised studies can directly be applied to challenging sequences. We employ different sensors with various resolutions and beamwidths in the experiments and show that GRAMME is sensor agnostic. Furthermore, we use game-theoretic approach to visualize the learnt feature space and illustrate the independent and uncorrelated failure modes of the proposed multimodal system, and show that GRAMME focuses on the relevant details in the environment. GRAMME is publicly available as an easy-to-use Python package^13^.

## Self-supervised artificial intelligence for all-weather ego-motion estimation

GRAMME is a deep learning-based self-supervised method that uses multiple sensors such as cameras, lidars and radars to estimate the ego-motion of AVs by reconstructing the three-dimensional scene geometry under diverse settings such as day, night, rain, fog and snow. GRAMME is sensor agnostic and designed to support sensors with various configurations in terms of resolution, beamwidth and field-of-view (Supplementary Note 5). GRAMME uses a novel differentiable view-reconstruction algorithm to incorporate the measurements of range sensors (for example, lidars and radars), mitigating the limitations of cameras (both monocular and stereo) under challenging conditions. The key supervision signal to train the neural networks for depth and pose prediction comes from the new view-reconstruction algorithm: given a multimodal input view of a scene, it reconstructs a new view of the scene captured from a different position. The visual-reconstruction algorithm uses the predicted per-pixel depth and ego-motion, whereas the range-reconstruction algorithm uses the predicted ego-motion and range measurements, both making use of multimodal masks. The spatial transformer module of GRAMME implements the view reconstruction in a fully differentiable manner compatible with the ego-motion, depth and mask prediction neural networks.

At a high level, GRAMME has a modular design to enable independent operation for each modality during both training and inference, which improves the robustness of the system to achieve a minimal risk condition^14^. Although we train the modules for depth, pose and mask predictions jointly, they can directly operate on the input frames separately from each other during test time, leading to independent and uncorrelated failure modes for the modules. Moreover, the modular design enables the performance gains achieved during multimodal training to be maintained at inference time even when the complementary modalities are partially or entirely unavailable. We use a reciprocal multimodal training technique to enhance the predictions on individual modalities, providing information flow across submodules. Furthermore, the range measurements of radar can directly capture strong patterns related to the geometry of the scene, whereas a simple colour value of camera pixels is associated with the geometry through an accurate depth estimation of the pixel. As the camera and radar measurements are perceptually different, we exploit a late multimodal deep fusion technique, which also facilitates the modular design. The multilayer perceptron-based late fusion layer uses the unaligned ego-motion predictions from multiple modalities to predict the ultimate motion. Due to the tight formulation of ego-motion and depth prediction, the multimodal fusion technique substantially improves the depth predictions as well. The fusion consists of two stages: first, the individual ego-motion predictions are used to reconstruct the corresponding camera and range views; second, the predictions of each modality are interchangeably used in the counterpart view-reconstruction algorithms for both visual and range reconstructions.

In the proceeding sections, we demonstrate the generalizability, data efficiency and interpretability of GRAMME in five different diverse settings such as day, night, rain, fog and snow. We qualitatively and quantitatively evaluate the state-of-the-art ego-motion estimation and depth prediction performance on multiple datasets, emphasizing the effect of modular design on individual modalities.

## Results

### Evaluation of model performance

We evaluated the depth and ego-motion prediction performance of GRAMME in five adverse settings such as day, night, rain, fog and snow using fivefold cross-validation. To quantitatively measure the generalization performance of GRAMME, we conducted an effective and reliable—yet rather challenging—cross-condition evaluation on the Robotcar dataset, enabled by the modular design of GRAMME. We trained the models on typical day sequences^15^ (training dataset) and directly evaluated them on more challenging conditions (night, rain, fog and snow)^16^ (test dataset). For each cross-validated fold, we randomly partitioned each public AV training dataset into a training set (80% of sequences), a validation set (10% of sequences) and a test set (10% of sequences). Each set contains the time-synchronized matching frames from each modality used for the training. The proportions of different settings (in terms of the number of frames) were kept constant in each set during partitioning. In each fold, we monitored the model’s performance on the validation set during training and used the validation set for model selection while the test set was held-out and referred to just once after training was complete to evaluate the performance of the model on day sequences. The final models are directly evaluated on the test datasets that contain adverse conditions never observed by the models during training.

### Multimodal, modular and generalizable depth prediction

**Fig. 2:**
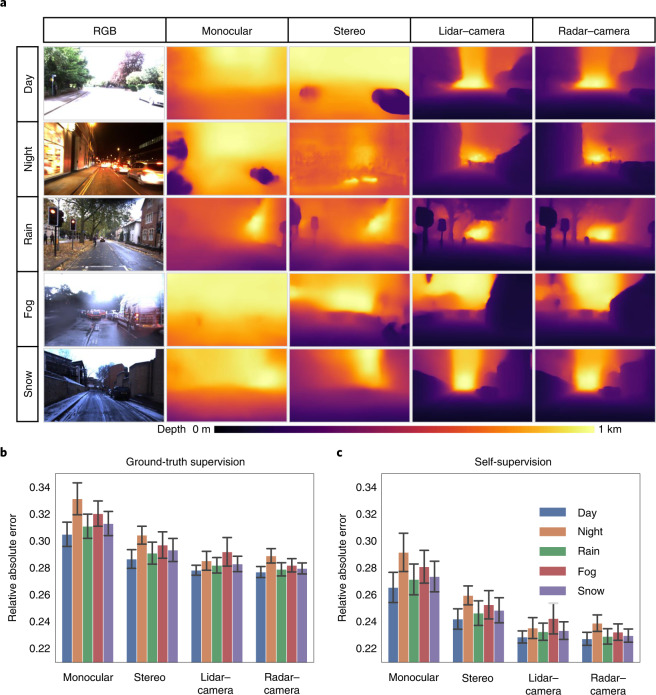
Multimodal, modular, and generalizable depth prediction performance. **a**, Qualitative results and sample test frames^16^ to visualize the generalization ability of GRAMME on depth prediction. We train each model using the day sequences in the training set and test them under diverse conditions to analyse the generalization performance. GRAMME successfully exploits the complementary aspects of the sensors. **b**, Comparatively weaker generalization performance of the supervised models. **c**, Quantitative results to compare the self-supervised generalization performance of GRAMME with respect to ground-truth supervision and intra-modality performance. The models trained only on camera are dramatically prone to failure in all of the challenging test conditions. Although lidar- and radar-based models provide qualitatively similar results and generally improve the overall performance, the model trained with radar provides greater immunity to precipitation. Error bars represent the depth prediction errors with respect to the ground truth. Camera fusion models employ the stereo setting.

**Extended Data Fig. 1:**
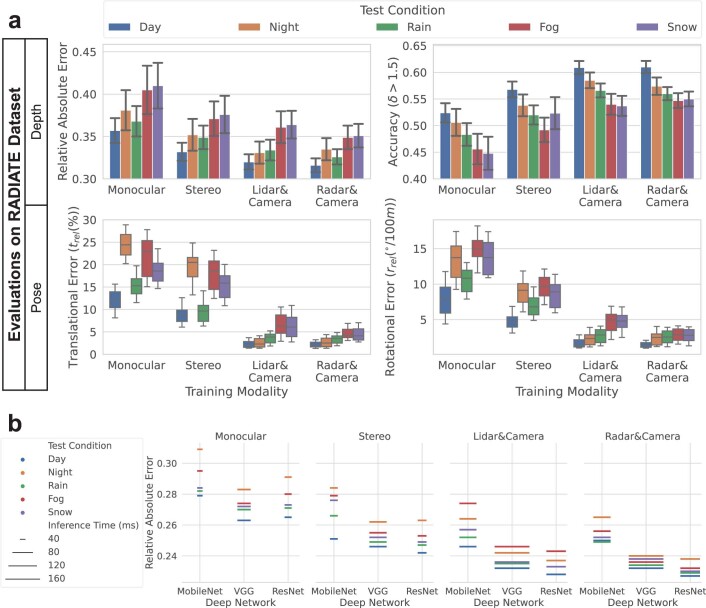
Performance on the RADIATE dataset, ablation study on the deep network, and run-time performance. (**a**) To evaluate the generalization of GRAMME to new datasets, we train, validate and test models on the publicly available RADIATE dataset^17^ that contains shorter sequences for ego-motion estimation with high variation in scene and structure appearance. We report the mean depth predictions errors with standard deviations and the distribution of motion prediction errors with quartiles. Although GRAMME performs well in terms of depth prediction and ego-motion estimation performance, we observe on this dataset that due to dense fog and heavy precipitation, the performance of the lidar&camera-based GRAMME model drops significantly compared to the day sequences. (**b**) As an additional ablation study, we replace the UNet network in the GRAMME modules with commonly used networks. The results show the basis for our choice of ResNet in the GRAMME architecture. We also report the run-time requirements in milliseconds, indicating the real-time capability of GRAMME on a consumer-grade GPU.

In the first set of experiments we analyse the depth prediction performance, which is a critical component of the self-supervision signal. GRAMME formulates multimodal ego-motion using a tight connection between depth prediction and ego-motion estimation to eliminate the need for the labelled data. The geometry-aware multimodal self-supervised architecture improves the generalization performance of the model to diverse conditions. Figure 2 shows the depth prediction performance for the model trained using: (1) a monocular camera, (2) a stereo camera, (3) a lidar–camera (stereo) and (4) a radar–camera (stereo). Note that we use only the day sequences in the training set for each training experiment on the modalities. Also, for each experiment, we use only the modalities labelled on the training modality column. Owing to GRAMME's modular design, the vision and range modules can make predictions directly and separately on the camera, lidar and radar inputs. To evaluate the generalizability of the depth module, we use the monocular sequences to test the depth prediction performance of the DepthNet module. The camera-only experiments also demonstrate the robustness of the system to sensor deficiencies. We also demonstrate the effect of external supervision by training the model with ground-truth pose information (as explained in the 'Datasets' section) following the same evaluation protocol. As shown in Fig. 2, ground-truth supervision reduces the generalization capability compared with self-supervision. Moreover, the relative multimodal performance of the supervised models is even worse than the self-supervised models. Although the camera-only self-supervised models are trained, validated and tested on day sequences and lead to overall performance improvement, challenging conditions involving glare and non-Lambertian surfaces still suffer from a considerable performance loss; however, the models trained on additional range sensors (that is, lidar and radar) are much more immune to such effects. Although stereo camera-based models are slightly better than their monocular counterparts, we have a similar observation on the other test conditions that the models trained only on camera are dramatically prone to failure. Moreover, although lidar- and radar-based models provide qualitatively similar results and generally improve the overall performance, the model trained with radar data provides greater immunity to precipitation. Under foggy conditions, the lidar measurements contribute relatively less to the generalization performance than to the other test conditions with higher error variance; this is caused by poorer measurements due to water droplets condensed on the sensor surface. On the other hand, the depth prediction of the model trained with lidar–camera fusion achieves better performance than the radar–camera model. As the lidar measurements are invulnerable to the lighting conditions and provide dense measurements, the model has an advantage over the radar-based version. GRAMME exploits the multimodal system design effectively, unlike the past work focusing mainly on either deep network architecture or objective function. The results show the benefits of multimodal fusion on depth prediction as an additional supervision signal, improving the generalization ability of the model under diverse settings. Moreover, we test the generalization performance of GRAMME to different datasets, repeating the same training, validation and test protocol on the publicly available RADIATE dataset^17^. We exhibit both the depth prediction and ego-motion estimation performance in Extended Data Fig. 1. Although the dataset contains shorter sequences with high variations in scene appearance and structure, GRAMME achieves remarkable domain adaptation performance on this challenging dataset (the observations on the RADIATE dataset is provided in Supplementary Note 6).

### Sensor-dependent masking, multisensory fusion and generalizable ego-motion estimation

**Fig. 3:**
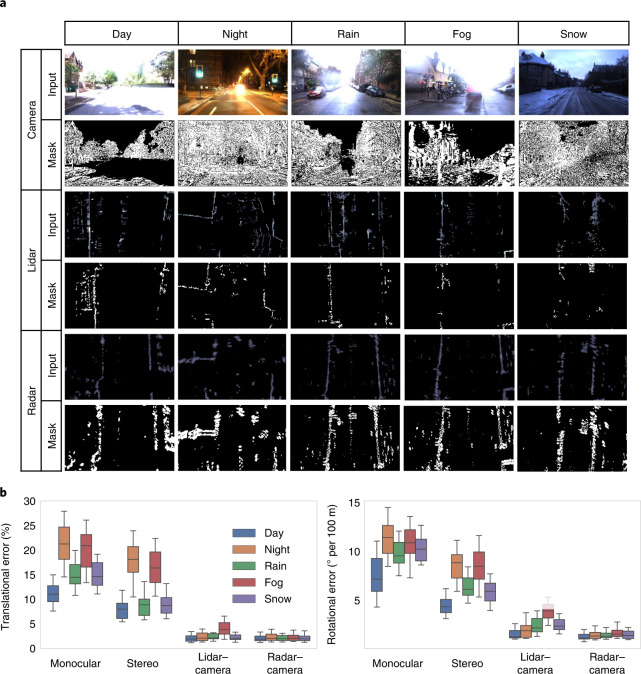
Sensor-dependent mask predictions and performance evaluations on generalizable multisensory ego-motion estimation under diverse settings. **a**, Illustration of sample frames^16^, multimodal measurements and the corresponding predicted masks. Each row shows a pair of input measurements and predicted masks of each modality. White and dark regions represent the valid and invalid points in the measurements to effectively capture the multisensory degradation resulting from both adverse weather and inherent sensor deficiencies, respectively. **b**, Multimodal performance evaluation on ego-motion estimation and multisensory fusion. The box plots show the median, first and third quartiles, as well as the minimum and maximum quartiles to show the errors in motion predictions. The error distribution in motion predictions in terms of the error quartiles are shown for translation and rotation components of motion for each modality. Sensor fusion greatler boosts the overall motion estimation performance.

View reconstruction provides the key supervision signal for the model training. In this set of experiments we investigate the effectiveness of the masking system as the major geometrically consistent element in the reconstruction. We then provide the overall generalization performance of the multisensor ego-motion estimation coupled with the masking system. As the view reconstruction is based on sampling from the adjacent frames, and occluded areas cannot be sampled by definition, reconstructed occlusion areas might corrupt the supervisory signal. The inherent heterogeneous radar artefacts such as ghost objects, phase and amplitude stability, speckle and saturation are other sources of inconsistency for view reconstruction (see Supplementary Note 3). Furthermore, the adverse weather conditions pose further challenges for camera and lidar that inhibit the underlying scene consistency assumptions. Poor weather introduces sharp intensity fluctuations in camera images, which degrade the consistency across frames. It is therefore important to detect the imperfect and unreliable regions in measurements and exclude them from the view reconstruction. GRAMME predicts a mask that is a combination of learnt and geometric masks to remove the invalid parts. The former is predicted by GRAMME's mask module, whereas the latter is based on the geometric inconsistency between consecutive multimodal frames that accounts for motion explanation, the nearly identical frames, and dynamic objects. We show that the predicted masks improve the performance of GRAMME by eliminating the imperfections on each modality. Figure 3 shows example frames and the corresponding mask predictions for each modality under challenging conditions. We use the stereo setting for the camera fusion models, which provides additional information due to binocular vision. To show that the masks eliminate the effect of unfavourable weather on each sensor, we trained the model using the individual modalities only. For example, intense glare caused by direct exposure to sunlight saturates most of the camera pixels and restrains the frame matching. The predicted camera mask captures the glaring regions and excludes them from the view reconstruction to prevent an incorrect consistency calculation that might corrupt the loss values computed during training. Similarly, although stereo camera provides binocular vision and is marginally less susceptible to occlusions than the monocular one, both camera types are still considerably prone to occlusions and poor visibility due to precipitation and weak illumination. For lidar, the reflections from the ground cause unreliable regions in the measurements that cannot be consistently matched across consecutive frames, which are detected and eliminated by the lidar masks. The mask also identifies false detections caused by fog droplets. On the other hand, although radar is more resistant to weather conditions, the radar measurements still suffer from the inherent artefacts discussed above. The radar masks seamlessly detect the imperfect measurements and filter them from the radar frames.

Following the same experimental protocol described above, we evaluate the generalization performance of the overall ego-motion estimation system. We show an ablation study of GRAMME in terms of the contribution of the fusion module to individual modalities and contribution of different sensors under unique test conditions. Figure 3 shows the translational and rotational errors of different ablation schemes, averaged over the day, night, rain, fog and snow test conditions. To evaluate the benefits of multisensor fusion, we train camera-only models in monocular and stereo settings. In separate training experiments, we fuse lidar and radar modalities with the stereo camera. As shown in Fig. 3, lidar–camera and radar–camera fusion significantly improves both the translational and rotational motion prediction performance compared with camera-only models. The GRAMME modal trained with lidar has notably higher errors in fog, showing the negative impact of fog on lidar data.

### Interpretability and dataset size-dependent performance

**Fig. 4:**
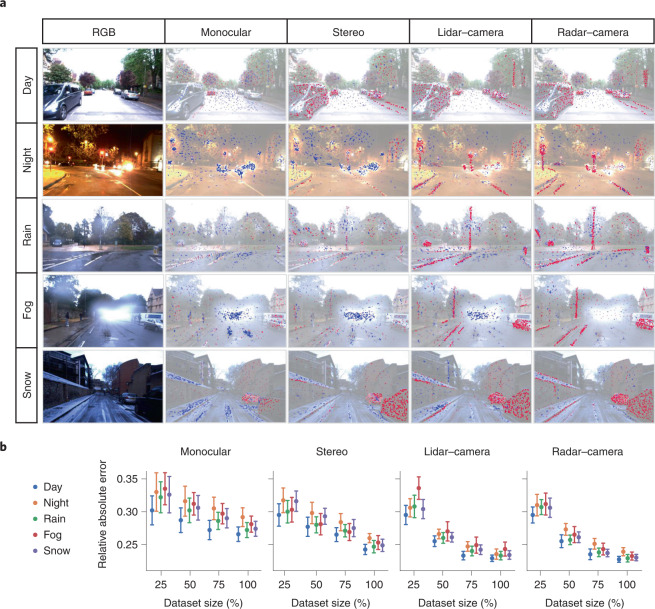
Interpretability and dataset size-dependent performance. **a**, A game-theoretic visualization of GRAMME to interpret the depth predictions based on the SHAP values for sample frames^16^. Pixels annotated by red points increase the depth prediction accuracy, whereas blue points lower the accuracy. The challenging conditions such as glare, poor illumination and adverse weather lead to concentrated blue regions around the occluded pixels. However, the training with lidar and radar data helps the model focus on more semantically invariant pixels across diverse test conditions, as visualized by the red points around static objects and road edges. The distribution of the values illustrates the independent and uncorrelated failure modes of the proposed multimodal system. **b**, Dataset size-dependent performance of GRAMME in terms of mean depth prediction error, with standard deviation with respect to the depth ground truth. Although the lidar–camera (stereo) and radar–camera (stereo) fusions improve the overall performance, access to minimal data (for example, only 25%) causes a worse performance than the camera due to the increased complexity of the model required for the multimodal architecture. On the other hand, despite the model complexity, the lidar and radar-based models achieve good performances (compared with the baseline approaches) with a dataset size of at least 50% in all test conditions.

Human interpretability of the trained self-supervised deep learning AV model not only serves to validate that the predictive basis of the model aligns with the intuitive geometry perception for depth and ego-motion prediction, but also promotes trust for end-users and liability for regulatory bodies^18,19^. We use a game-theoretic approach to visualize the contribution scores of each pixel by decomposing the output prediction of the DepthNet module on a specific input by back-propagating the contributions of all neurons in the network to every feature of the input frame. The visualization is based on Shapley additive explanations (SHAP)^20^ that assigns each feature an importance value based on SHAP values for a particular prediction. GRAMME models make the multimodal predictions by first identifying and aggregating regions in the vision and range sensor measurements that are of high predictive importance (high SHAP values, red) while ignoring regions of low relevance (low SHAP values, blue); see Fig. 4 for the visualization of SHAP values on sample multimodal inputs for different training modalities. Although the higher SHAP values on the camera-only models are concentrated around static objects, they are usually scattered across input images. Besides, the lower SHAP values are more frequent than the higher values and concentrate around the imperfections on the input such as glare and occlusions. However, when the model is trained with lidar and radar sensors, the SHAP values focus on the object region with geometric structures (for example, cars and static objects), and the layout (house silhouette and road boundaries). The fusion model focuses on the structural representations that reflect essential information for depth estimation, which is semantically more consistent between various unfavourable conditions such as night, rain, fog and snow. Note that the fusion models are trained with multiple modalities, but the tests are conducted on the camera depth prediction without access to the data from the additional sensors. The camera fusion models refer to the stereo setting. Although the DepthNet module trained with the camera struggles to find consistent and depth-related points, the fusion of additional sensors that are more resistant to environmental changes helps the DepthNet focus on geometric structure and object boundaries even when it does not have access to the lidar and radar data at test time.

Motivated by the inadequacy of accurate ground-truth data in diverse, multimodal datasets at scale, we evaluated the performance of GRAMME with sequentially sampled subsets of training data under different test conditions (25%, 50%, 75% and 100%) while keeping the validation and test sets the same to investigate the dependency of the model’s performance on the amount of training data available. Figure 4 shows the relative absolute error for multimodal depth prediction in diverse settings, visualizing the median, first and third quartile of errors. When supervising GRAMME models with the smaller, sampled subsets of training data, we observed that the number of frames required to achieve satisfactory performance (with respect to the baseline monocular performance of MonoDepth2; ref. ^21^) varies depending on the modality and the test condition. For example, fusion models achieve good performance with a dataset size of at least 50% in all test conditions. However, the model trained with cameras needs at least 75% of the training dataset. Notably, the performance of fusion models might deteriorate with access to very limited data (for example, with only 25% of the dataset). The increased complexity needed to implement the multimodal architecture makes the model more data-dependent than those with single modalities. Furthermore, although radar–camera fusion provides more immunity to adverse weather than lidar–camera fusion, the latter performs relatively well under the poor illumination in the night sequences. Both lidar and radar modalities are not affected by the illumination, but the lidar model utilizes the dense measurements of the lidar sensor and achieves better performances, accordingly.

## Discussion

We showed that GRAMME addresses five key challenges in autonomous driving. The first is multimodal self-supervision: we trained models with self-supervision using only the sensor measurements captured by the camera, lidar and radar sensors. We formulated a differentiable view-reconstruction algorithm to create a supervisory signal from range scanning sensors (that is, lidar and radar). We demonstrated that multimodality improves the robustness of the model to poor illumination and adverse weather, while self-supervision eliminates the need for cumbersome ground-truth collection and improves the generalization capability compared with supervised approaches. A possible explanation for the poor generalization performance of the supervised models is that they are optimized to learn the relationship between the input frames and the ground truth rather than the underlying geometry. We also demonstrated that multimodal self-supervision achieves state-of-the-art depth reconstruction and ego-motion estimation results compared with the established self-supervised approaches. Although radars provide a reliable complementary perception, the imaging radars are still sparse and the resolutions are limited. We argue that the development of higher resolution radars in three-dimensions will be a milestone enabler for all-weather AVs. The second challenge is modularity: we trained models using different modalities in various settings. We showed that the modules could be trained and validated with partial availability of the intermediate outcomes and the other modules, resulting in a more robust system under diverse settings. We further showed that the modules could transfer the improved capabilities acquired during multimodal training to test time even when the other modalities are partially or completely unavailable at test time, leading to independent and uncorrelated failure modes. We argue that modularity is an essential capability to achieve a minimal risk condition, improving the safety of AVs in case of hardware or software failures of the components. Although a unitary design with tight connections might result in performance gains, it should not come at the cost of safety. The third challenge is generalizability: unlike most past self-supervised studies, we also focused on generalization to all of the weather conditions. We trained the model using only day sequences in the training set and directly evaluated it against the other conditions. Camera-only models showed poor generalization due to the degraded performance of cameras under challenging conditions; however, we showed that models trained with multiple modalities (that is, lidar and radar) achieve a substantial performance boost in terms of generalizability to unseen conditions during training. Although we use a diverse dataset including several difficult conditions that AVs might commonly face during regular operation, it is beyond feasible to cover all kinds of adverse conditions in an AV dataset. Research on generalization performance under unfavourable weather conditions is thus particularly crucial for the development of AVs. Furthermore, the existing public AV datasets in the literature cover a wide range of conditions, but they do not extensively cover extreme conditions such as heavy downpours and large snowfalls, which is a limiting factor in evaluating the generalization capability. Fourth, interpretability: we demonstrated that our models are interpretable and capable of capturing semantically and geometrically consistent regions. We visualized the extracted features using SHAP values and observed that the camera-only model struggles to focus on consistent regions across frames. On the other hand, multimodal training helps the model to capture more consistent areas that are interpretable by humans. Although deep learning models are heavily deployed in an AV software stack, interpretability remains a considerable challenge due to the lack of insightful and lucid interpretability approaches to analyse the complex deep learning architectures. Finally, data efficiency: our quantitative experiments and comparative analysis demonstrated that GRAMME models trained with multiple modalities achieve satisfactory results compared with baseline methods, even with dataset sizes limited by up to 50%, despite the increased complexity. However, the specific inherent vulnerability of sensors (such as lidar in fog) might deteriorate the performance with minimal data availability (for example, 25% of a dataset). For depth and ego-motion estimation in adverse weather, we believe that the diversity and accuracy of ground truth in the existing public datasets are still insufficient, which is likely to constitute a limiting factor; data-efficiency analysis is therefore important to understand how sensitive is the performance of a deep learning model to the availability of additional data.

The key aspects discussed above address a critical issue of AVs: the ability to know precisely where they are on the map. Core AV components such as prediction and planning rely on this localization ability. In this study we showed that robust and accurate ego-motion estimation provides a complementary solution to localization and is a critical component of autonomous driving to achieve safety and reliability under adverse conditions. The high level of location accuracy provided by GRAMME enables AVs to reliably understand their environment and make safer decisions. We demonstrated that the complementary and redundant perception that AVs gain from multiple sensors improves the reliability of vehicles in challenging situations, especially in unfavourable weather conditions. Furthermore, the self-supervised aspect of GRAMME enables artificial intelligence systems deployed on AVs to learn localization from orders of magnitude more data, which is important to quickly recognize and understand new driving conditions. We believe AV technologies should meticulously involve these fundamental aspects to achieve safe and reliable autonomous driving.

In terms of future directions, the presented technology can be further improved in several directions. For example, the signal-to-noise ratio of range sensors can be integrated into the masking component of GRAMME, providing an additional physical source of confidence for the measurements. Moreover, the Doppler measurements from radars can help the model better distinguish dynamic and static objects in the scene, enabling a more consistent geometric and semantic understanding of the environment. Moreover, GRAMME as a learning-based approach can be extended to higher level learning schemes of autonomous driving such as lifelong and continual learning, resulting in AVs that continuously and collaboratively improve autonomous driving artificial intelligence.

## Methods

### GRAMME

**Extended Data Fig. 2:**
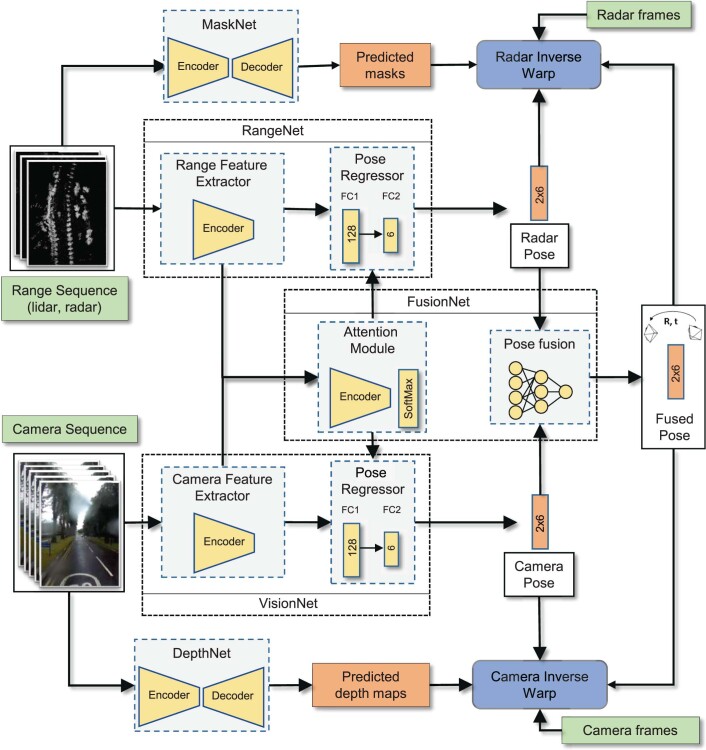
Detailed architecture design of the proposed geometry-aware, multimodal, modular, interpretable, and self-supervised ego-motion estimation. The modules consisting of encoder and decoder networks are based UNet architecture with skip connections. Feature extractors with an encoder are based on the ResNet18 network visualized on sample input^17^. *FC* layers represent fully connected layers. Pose fusion network is a multilayer-perceptron. As part of the spatial transformer module, the inverse warp algorithm re-uses the input target frames to calculate the reconstruction loss. Camera input can be set to contain more frames than the range sensor due to the higher fps rate. The fused pose is the final output, optimized in a self-supervised manner without ground-truth with respect to the intermediate pose and depth predictions.

GRAMME is a self-supervised deep learning framework designed to robustly estimate the ego-motion and depth map for an AV under diverse settings. GRAMME follows an end-to-end design and leverages data-driven learning to combat the inherent limitations of conventional and state-of-the-art ego-motion estimation methods. GRAMME demonstrates the feasibility of multimodal odometry under adverse weather conditions and proposes a multisensor fusion framework, resulting in a robust ego-motion estimation system. The standard self-supervised ego-motion prediction is based on monocular camera, and it consists of two joint stages^22,23^. The first stage predicts a depth map for a given camera frame, whereas the second stage predicts ego-motion between two consecutive camera frames. Given the ego-motion and depth predictions, a spatial transformer algorithm reconstructs the target camera frame from the source frames. The spatial transformer module builds on the idea presented by Jaderberg et al.^24^, explicitly allowing the spatial manipulation of multimodal data within the network. The reconstruction quality establishes the supervisory signal to optimize the neural network. GRAMME builds on the self-supervised training idea and describes a multimodal architecture to promote complementary sensor behaviours, yielding a robust ego-motion estimation for AVs under diverse settings such as day, night, rain, fog and snow. GRAMME introduces a novel differentiable range-reconstruction algorithm for range frames (that is, lidar and radar) as part of its multimodal spatial transformer that is adaptable to the back-propagation during training of the deep learning architecture. The RangeNet module uses two consecutive range frames to predict the ego-motion of AV, whereas MaskNet predicts the reliable regions in individual frames. Given the ego-motion and mask predictions, the spatial transformer algorithm uses the source frames to reconstruct the target range frames. To exploit the complementary information obtained from different sensors, GRAMME proposes a fusion method that consists of the FusionNet layer and cross-modal training technique. The novel fusion method enables information flow across different modalities due to the joint training technique, improving the robustness of individual modalities. Extended Data Fig. 2 shows the details of the architecture.

#### Problem definition

Each loosely time-synchronized triplet of consecutive camera ($<{{{{\bf{I}}}}}_{s,i-1}^{c},{{{{\bf{I}}}}}_{t,i}^{c},{{{{\bf{I}}}}}_{s,i+1}^{c}>$), lidar ($< {{{{\bf{I}}}}}_{s,i-1}^{l},{{{{\bf{I}}}}}_{t,i}^{l},{{{{\bf{I}}}}}_{s,i+1}^{l} >$) and radar ($< {{{{\bf{I}}}}}_{s,i-1}^{r},{{{{\bf{I}}}}}_{t,i}^{r},{{{{\bf{I}}}}}_{s,i+1}^{r} >$) frames in the training set (${{{\mathcal{I}}}}={{{{\mathcal{I}}}}}_{\mathrm{c}}\cup {{{{\mathcal{I}}}}}_{\mathrm{l}}\cup {{{{\mathcal{I}}}}}_{\mathrm{r}}$) represents a single data point at time index *i* with unknown ego-motion and depth map of the camera source **I**_s_ and target **I**_t_ frames. Our goal is to estimate **T**, where the pose **T**_*t*→*s*_ = [**R**∣**t**] ∈ **SE**(3) is a transformation between the target (*t*) and source (*s*) frames with rotation matrix **R** and translation vector **t**. Although the standard commercial radars are two-dimensional sensors, we formulate our problem in **SE**(3) to enable compatibility with other three-dimensional sensor modalities. Unlike existing self-supervised radar approaches^25^, GRAMME directly predicts the pose between the consecutive frames without imposing strong motion prior factors.

#### Camera module

The camera module consists of two networks. DepthNet uses UNet style skip connections^26^ to predict per-pixel depth map **D** of a given RGB image. In parallel, VisionNet follows ResNet18 architecture to predict the relative pose **T**_*t*→*s*_ between source and target RGB images $< {{{{\bf{I}}}}}_{s}^{c},{{{{\bf{I}}}}}_{t}^{c} >$. We use the predicted depth and pose values in the spatial transformer algorithm to create a supervisory signal based on perspective projection. However, photometric error supervision alone is ambiguous, especially in low-textured regions due to the multiple matches with one pixel. To prevent depth ambiguity due to incorrect pixel matches in low-textured and occluded areas, we apply a regularization:

1 $${L}_{\mathrm{s}}(D,2)=\mathop{\sum}\limits_{{x}_{t}}\mathop{\sum}\limits_{d\in x,y}| {\nabla }_{d}^{2}D({x}_{t})| {\mathrm{e}}^{-\alpha | {\nabla }_{d}I({x}_{t})| }$$

*L*_s_(*D*, 2) is a second-order spatial depth smoothness term that penalizes the divergence of the depth prediction gradients along both the *x* and *y* directions^22^. The regularization encourages the alignment of the depth values in the planar surface in the absence image gradients. For multiview projection between multiple camera views, let *D*(*x*_*t*_) denote the depth value of the target image at coordinate *x*_*t*_, and **K** be the camera intrinsics matrix. Assume a rigid transformation **T**_*t*→*s*_ is the relative pose from the target view to source view, and *h*(*x*) is the homogeneous coordinates given *x*. The perspective projection to find corresponding pixels in the source view can be formulated as,

2 $$D({x}_{s})h({x}_{s})={{{\bf{K}}}}{{{{\bf{T}}}}}_{target\to source}D({x}_{t}){{{{\bf{K}}}}}^{-1}h({x}_{t})\,$$

and the image coordinate *x*_*s*_ can be obtained by de-homogenization of *D*(*x*_*s*_)*h*(*x*_*s*_); *x*_*s*_ and *x*_*t*_ are therefore a pair of matching coordinates in the source and target views, and the similarity between the two can be compared to validate the correctness of structure. Given the pixel-wise matching pairs in ${{{{\bf{I}}}}}_{t}^{c}$ and ${{{{\bf{I}}}}}_{s}^{c}$, we can reconstruct a target view ${\hat{{I}_{s}}}^{c}$ from the given source view as described in ref. ^27^, and calculate the final camera objective using the photometric error ${L}_{\mathrm{c}}={L}_{\mathrm{p}}({{{\bf{M}}}},{\hat{{{{\bf{I}}}}}}_{s}^{c},{\hat{{{{\bf{I}}}}}}_{t}^{c})+{\lambda }_{{s}}{L}_{\mathrm{s}}({{{\bf{D}}}})$ following the camera masking method offered in ref. ^22^. The camera module is applicable to monocular and stereo cameras by exploiting the left–right consistency^21^.

#### Range module

The range module is designed to predict ego-motion from radar and lidar measurements that are represented by a bird’s-eye view in Cartesian coordinates, consisting of two feature extractor networks based on ResNet18 followed by two fully connected layers to regress the relative pose. RangeNet predicts the relative pose **T**_*t*→*s*_ between source and target frames <**I**_*s*_, **I**_*t*_>, whereas MaskNet individually predicts a mask **M** in parallel to detect the consistent regions in the frames. Finally, our view synthesis algorithm reconstructs the target view using the predicted pose and mask.

#### View synthesis for range sensors

Given a source **I**_*s*_ and target **I**_*t*_ views in Cartesian coordinates for radar and lidar measurements, we use the relative predicted pose **T**_*t*→*s*_ between the views to reconstruct a target view ${\hat{{I}_{s}}}^{c}$ through bilinear interpolation. To reconstruct the value of ${\hat{I}}_{s}({x}_{t})$ from the value of *I*_*s*_(*x*_*s*_), we use a differentiable bilinear sampling mechanism similar to the photometric approaches^24^, linearly interpolating the values of the four-pixel neighbours ${{{\mathcal{N}}}}=$ (top-left, top-right, bottom-left and bottom-right) of *x*_*s*_ to approximate *I*_*s*_(*x*_*s*_), that is, ${\hat{I}}_{s}({x}_{t})={I}_{s}({x}_{s})={\sum }_{i,j\in {{{\mathcal{N}}}}}{w}^{ij}{I}_{s}({x}_{s}^{ij}),$ where *w*^*ij*^ ∝ ∣*x*_*s*_ − *x*_*t*_∣, and ∑_*i*,*j*_*w*^*ij*^ = 1; then, given the Lambertian and a static rigid scene assumptions, we can calculate the average intensity error to refine the predicted relative pose. However, this assumption is not always true because of dynamic objects and sensor deficiencies, which might be further violated under adverse weather. We introduce a consistency mask **M** to compensate for the regions violating the assumption. Formally, the masked intensity loss for lidar (*L*_l_) and radar (*L*_r_) is,

3 $$\begin{array}{l} {L}_{\mathrm{l,r}}({{{\mathbf{M}}}},{\hat{{{{\mathbf{I}}}}}}_{s},{\hat{{{{\bf{I}}}}}}_{t})= {\mathop{\sum }\limits_{s=1}^{S}}{\mathop{\sum}\limits_{{x}_{t}}{{{{\mathbf{M}}}}}_{s}}({x}_{t})| {I}_{t}({x}_{t})-\hat{{I}_{s}}({x}_{t})| ,\\ {\text{such that}}\,{\forall } x_{t},s\,\,{\bf{M}}_{s}(x_{t})\in [0,1]\end{array}$$

where ${\{{\hat{I}}_{s}\}}_{s = 1}^{S}$ is the set of reconstructed source views, {**M**_*s*_} is a set of consistency masks, and **M**_*s*_(*x*_*t*_) ∈ [0, 1] provides a weight on the error at *x*_*t*_ from source view *s*. The range-reconstruction algorithm is summarized in Algorithm 1. Moreover, the explainability mask has a trivial solution in this formulation, assigning all mask values to zero. We apply a regularization term to encourage non-zero masks to prevent the saturation in the network activation, using a cross-entropy loss for the predicted masks:

4 $${L}_{\mathrm{m}}({{{\bf{M}}}})=-\mathop{\sum}\limits_{s}\mathop{\sum}\limits_{{x}_{t}}\log P({{{{\bf{M}}}}}_{{{{\bf{s}}}}}({x}_{t})=1).$$

In the bird’s-eye view, vehicles and large objects occupy smaller areas compared with the front-view. For example, a vehicle with an average size of 2.5 × 5.1 m occupies only a 13 × 26 pixels area with an input resolution of 0.2 m. Downsampling the bird’s-eye view map through the encoder makes the region-wise features vulnerable to quantization errors in the subsequent mask generator; thus, GRAMME upsamples the coarse-grained feature map via a transposed convolution layer (decoder) and concatenates the output with the fine-grained feature map with skip links, following the UNet design^26^.

#### Multimodal fusion

GRAMME introduces a self-supervised fusion approach that involves an attention module, a fusion network and a training technique. The features extracted from range and camera modules are used in an attention module to create weighted features. Lidar and radar features are not always equally important, and their contributions to final pose prediction should be weighted accordingly. We extract a weight vector from the concatenated features through a ResNet18-based encoder followed by a fully connected layer and a SoftMax layer to predict the importance weights between [0, 1] for each input feature. The pose regressors then use the weighted features to predict the relative pose. FusionNet uses the unaligned relative pose values predicted by VisionNet and RangeNet, and predicts the ultimate ego-motion without any correction using the extrinsic calibration among the sensors. Furthermore, during multimodal training, we impose a cross-modal fusion loss *L*_f_, in which we use the fused pose in the range-reconstruction algorithm and calculate the reconstruction error. The cross-modal training technique not only increases the robustness of ego-motion prediction but also improves the predictions from the other modalities, such as depth prediction.

#### Training details

During training, the triplet frames in the training set are randomly sampled and provided to the model using a batch size of 16. We augment the lidar and radar scans with a random rotation around the vehicle centre by an angle in [−10, 10]^∘^ because a large fraction of the AV datasets consists of either driving straight or waiting in traffic. The total loss for a given sequence *L* is the sum of the individual modality losses and masking loss with optional scaling factors *λ*_*c*_ for the camera component and *λ*_*m*_ for the mask component of the loss. The final learning objective is given by:

5 $$\begin{array}{ll}L({{{\bf{D}}}},{{{\bf{T}}}},{{{\bf{M}}}})=&{L}_{\mathrm{l,r}}({{{\bf{M}}}},{\hat{{{{\bf{I}}}}}}_{s},{\hat{{{{\bf{I}}}}}}_{t})+{\lambda }_{c}{L}_{\mathrm{c}}({{{\bf{D}}}},{{{\bf{T}}}},{{{\bf{M}}}})\\ &+\,{\lambda }_{m}{L}_{\mathrm{m}}({{{\bf{M}}}})\end{array}$$

Given the objective functional, the photometric and intensity error is back-propagated to depth, pose and mask networks by applying the spatial transform operation to supervise the learning process. We used *λ*_*c*_ = 30 and *λ*_*m*_ = 1 for all experiments. Our models are implemented in PyTorch^28^, trained for at least 50 epochs until 200 epochs using Adam^29^ unless an early stopping criterion is met, which was chosen using the validation set explained in Section Results. The validation loss is monitored each epoch, and early stopping is triggered when it has not decreased from the previous low for over five consecutive epochs. The saved model with the lowest validation loss is then evaluated on the test set. We use Adam optimizer using a learning rate of 1 × 10^−4^ with an L2 weight decay of 1 × 10^−5^.

**Algorithm 1. View reconstruction for range sensors**

 *m*, *n* ← Height, Width ⊳Input dimensions

 **C** ← Stack((1, 2, ... , *m*), (1, 2, ... , *n*)) ⊳ Pixel coordinates in homogenous form

 **function** InverseWarp(**I**_*s*_, **p**_*s*_, **M**_*s*_)

 **T**_*t*→*s*_ = Rodrigues2TransformationMatrix(**p**_*s*_)

 $$\tilde{{{{\bf{C}}}}}={{{{\bf{T}}}}}_{t\to s}{{{\bf{C}}}}$$ ⊳ Transformed points

 $\tilde{{{{\bf{C}}}}}=$ Normalize$(\tilde{C})$ ⊳ Normalized pixel coordinates in [−1, 1]

 ${\hat{{{{\bf{I}}}}}}_{s}=$ BilinearSample$(\tilde{{{{\bf{C}}}}},{{{{\bf{I}}}}}_{s})$ ⊳ Reconstructed frame

 **if** **M**_*s*_! = None **then**

  ${\tilde{{{{\bf{M}}}}}}_{s}=$ BilinearSample$(\tilde{{{{\bf{C}}}}},{{{{\bf{M}}}}}_{s})$ ⊳ Reconstructed mask

 **else**

  $${\tilde{{{{\bf{M}}}}}}_{s}=1$$

 **return** $${\hat{{{{\bf{I}}}}}}_{s},{\tilde{{{{\bf{M}}}}}}_{s}$$

### Datasets

We design GRAMME for multiple modalities such as camera, lidar and radar, exploiting the complementary features of each sensor under diverse settings. Although there are several publicly available AV datasets, they typically employ sparse lidar and radar sensors, useful for object detection, but not for three-dimensional perception. Moreover, they mostly consist of common daytime conditions only, which is not enough to evaluate the performance of an AV module under diverse conditions. Under these requirements, we conduct our experiments on the Oxford Robotcar^16^ and the RADIATE^17^ datasets. Figure 1 shows samples from the RADIATE dataset, whereas Figs. 2–4 show samples from the Robotcar dataset. We follow the same coordinate reference systems as suggested by the authors of these datasets to achieve a standard evaluation setup for comparisons. The Oxford Radar Robotcar (ORR) dataset is collected by a vehicle equipped with a NavTech CTS350-X radar, and two co-located Velodyne HDL-32E lidars at the roof centre. The dataset contains the merged point clouds of the lidars, providing the ground truth for depth maps. The authors of the Oxford Radar Robotcar Dataset^15^ include visual odometry and loop closures into a large-scale optimization of their GPS/INS system to provide the ground-truth trajectory. The ORR radar scans the 360° field of view at an angular step of 0.9° every 0.25 s, and the lidar at a step of 0.33° every 0.05 s. The dataset provides the radar and lidar scanning results transformed into a two-dimensional intensity map and three-dimensional point cloud, respectively. Both sensors share the same coordinate origin. The ORR dataset contains 8,862 samples, which are split into 7,090 for training, 886 for validation and 886 for testing, without geographic overlapping. The dataset includes thirty-two traversals around 280 km of driving in total. We also evaluate our model on an earlier version of the ORR dataset, Oxford Robotcar dataset^16^ that contains the same lidar and camera sensors except for the radar. This version is collected in a period of one year in Oxford, and around 1,000 km in total. We use several sensors attached to the Oxford RobotCar: a Bumblebee XB3 stereo camera, and a SICK LD-MRS three-dimensional lidar with a drastically limited field of view, unlike the lidar on the newer version of the dataset. Within this configuration, the lidar and stereo camera yield a data stream on 11 fps and 16 fps, respectively. On the other hand, although the RADIATE dataset^17^ is collected mainly for object detection, it is still an interesting dataset as it contains shorter sequences with high variation in scene appearance compared to the Robotcar dataset. The RADIATE dataset involves a ZED stereo camera at 15 fps, which is protected by a waterproof housing under extreme weather conditions. The images might have severe blur, haze or might be obstructed due to raindrops, dense fog or snow flakes. A 32 channel, 10 Hz, Velodyne HDL-32e LiDAR gives 360° coverage. The lidar data can be missing and noisy since the signal can be severely attenuated and back-scattered by intervening fog or snow. The RADIATE dataset adopts the same radar as the Robotcar dataset: the Navtech CTS350-X radar that is a scanning radar providing 360° high-resolution range-azimuth images without Doppler information. The radar is set to 100 m maximum operating range with 0.175 m range resolution, 1.8° azimuth resolution. For both datasets, we follow the original implementation of the authors for the conversion of radar frames from polar to Cartesian coordinates and bird’s eye view projection of lidar frames. Figures 2–4 illustrate the large differences in weather conditions. Between the day and snow conditions, there was significant dissimilarity in visual appearance. For example, most of the lane lines are barely visible during the snow.

### Comparative analysis and ablation study

#### Ego-motion estimation

We compare the ego-motion estimation performance of GRAMME with baseline methods, shown in Extended Data Table 1. In accordance with the baselines, we use the same spatial cross-validation setting suggested by Barnes et al.^30^, and report our results using the KITTI odometry metrics ^31^, which average the relative position and orientation errors over every sub-sequence of length (100 m, 200 m, … , 800 m). Evaluation of ego-motion estimation techniques based on full global trajectory end-points is misleading because a large motion error in the earlier trajectory points leads to substantial errors in the end-points. We thus use trajectory segments to analyse rotation and translation errors, following the standard evaluation benchmarks described in ref. ^31^ to allow for deeper insights into the qualities and failure modes of motion prediction. To provide a comprehensive analysis, we evaluate the competing approaches using the camera, lidar and radar modalities. To provide a fair comparison, we report both the individual and fused modalities for GRAMME. We evaluate the proposed approaches in three different settings as grouped in Extended Data Table 1: camera, lidar and radar ego-motion estimation. GRAMME models trained with additional data are indicated in parentheses, where the camera fusion refers to the stereo setting. Regardless of the training strategy of each method, each competing approach receives the same input without additional data at test time. Although we use exactly the same GRAMME camera model for each experiment, the model trained with additional data outperforms the competing approaches by a considerable margin, proving the effectiveness of the proposed multimodal approach. Besides, we independently report the performance of the competing approaches from Adolfsson and colleagues^32^. For camera-based evaluation, we compare GRAMME in monocular and stereo settings with the visual odometry method used in the Robotcar dataset, which employs an extensive number of features at the cost of a high computational burden^33^. We also compare our method with ORB-SLAM2^34^, which loses the track and fails on the sequences shown in Extended Data Table 1. On the other hand, GRAMME in stereo setting successfully completes all of the test sequences and substantially outperforms the baselines. Similar to ORB-SLAM2, the lidar-based approaches LOAM^35^, Lego-LOAM^36^ and SuMa^37^ fail to finish the whole sequences or rapidly deviate due to the challenging dynamics. Similar to LOAM, Lego-LOAM is tightly linked to the mapping and failed to perform odometry without the mapping module. We therefore report the results for Lego-LOAM with the mapping module enabled. The GRAMME lidar model outperformed the proposed approach on the full field-of-view lidar setting. Hence we report their results up to the point where they lose tracking. Note that we project the six degrees-of-freedom pose predictions provided by the vision and lidar approaches onto the XY plane for evaluation. It can be seen that the proposed GRAMME can achieve comparable or better localization accuracy with enhanced robustness. On the other hand, although the GRAMME model trained on a low-cost lidar (for example, SICK LD-MRS 3D LIDAR 85^∘^ HFoV) and labelled as narrow in the table shows worse performance than the full field-of-view alternative, it successfully completes all the test sequences. The poor performance is caused by the limited measurement capability of the sensor that is mainly designed for obstacle detection at a short range within a limited view. SuMa achieves similar performance to GRAMME in terms of odometry accuracy. The main reason is that GRAMME is designed for bird’s-eye-view images, whereas SuMa specializes in front-view lidar inputs. Specifically, SuMa constructs and reserves a surfel-based map of the environment, which embodies dense information for front-view lidar input, but sparse and isolated information for bird’s eye view images. However, the GRAMME model based on lidar and camera shows a better performance with a noticeable margin, demonstrating the effectiveness of the fusion approach. Moreover, we compare our radar-based GRAMME model with state-of-the-art radar odometry methods provided by Cen et al.^38^, Barnes et al.^30^ and Hong et al.^39^. The results show that GRAMME radar-only model surpasses the performance of both geometry- and learning-based approaches. Besides, GRAMME exceeds the performance of the supervised radar odometry approach^30^ without any need for ground-truth supervision, which indicates the advantage of GRAMME deployment in regions where a source of high-quality location information is unavailable such as a GPS/INS system.

#### Depth prediction

We comparatively evaluate the depth prediction performance of GRAMME using the error and accuracy metrics that were initially proposed by ref. ^40^ and widely adopted in the literature. Also, as a convention in the competing approaches, we evaluate the performance of depth prediction capped at 60 m as the measures without threshold can be sensitive to the great errors in depth caused by prediction errors at small disparity values. Although DepthNet predicts the dept maps within [0 − 1 km] range for better visualization, the reported errors are capped to achieve a common evaluation criteria. Note that the range of the depth prediction formulated in equation (2) is theoretically not limited. The error and accuracy metrics used in the evaluations are defined as:

$$\begin{array}{rcl}{\mathrm{AbsRel}}& \equiv & \frac{1}{| {{\Omega }}| }\mathop{\sum}\limits_{(x,y)\in {{\Omega }}}\frac{| D(x,y)-{D}^{{{{\rm{gt}}}}}(x,y)| }{{D}^{{{{\rm{gt}}}}}(x,y)}\\ {\mathrm{SqRel}}&\equiv & \frac{1}{| {{\Omega }}| }\mathop{\sum}\limits_{(x,y)\in {{\Omega }}}\frac{| D(x,y)-{D}^{{{{\rm{gt}}}}}(x,y){| }^{2}}{{D}^{{{{\rm{gt}}}}}(x,y)}\\ {\mathrm{RMSE}}&\equiv & \sqrt{\frac{1}{| {{\Omega }}| }\mathop{\sum}\limits_{(x,y)\in {{\Omega }}}| D(x,y)-{D}^{{{{\rm{gt}}}}}(x,y){| }^{2}}\\ {\mathrm{RMSElog}}&\equiv & \sqrt{\frac{1}{| {{\Omega }}| }\mathop{\sum}\limits_{(x,y)\in {{\Omega }}}| \log D(x,y)-\log {D}^{{{{\rm{gt}}}}}(x,y){| }^{2}}\\ {\log }_{10}&\equiv & \frac{1}{| {{\Omega }}| }\mathop{\sum}\limits_{(x,y)\in {{\Omega }}}| \log D(x,y)-\log {D}^{{{{\rm{gt}}}}}(x,y)| \\ {\mathrm{Accuracy}}& \equiv & \% \, {\mathrm{of}} \, D(x,y) \, {\mathrm{s.t.}} \,\delta \doteq \max \left(\frac{D(x,y)}{{D}^{{{{\rm{gt}}}}}(x,y)},\frac{{D}^{{{{\rm{gt}}}}}(x,y)}{D(x,y)}\right) < \tau \end{array}$$

*D*(*x*, *y*) is the predicted depth at (*x*, *y*) ∈ Ω and *D*^gt^(*z*, *y*) is the corresponding ground truth. We use the most common three different thresholds *τ* (1.25, 1.25^2^ and 1.25^3^) in the accuracy metric. Since the monocular camera lacks the absolute scale, we multiply the monocular depth predictions by a scaling factor, *s*, that matches the median with the ground-truth depth map to solve the scale ambiguity issue, that is, *s* = median(**D**^gt^)/median(**D**). The depth prediction results in terms of those metrics are shown in Extended Data Table 2. We evaluate the depth prediction performance of the competing approaches under diverse settings such as day, night, rain, fog, and snow, following the same training and test protocol. We use Monodepth2 (ref. ^21^) as a baseline, which is the most similar architecture to the camera module of GRAMME. We train, validate and test it using the same dataset split as GRAMME. Although Monodepth2 achieves comparable results in day sequences, it performs poorly in reduced visibility conditions due to the occlusions and low lighting. Since the camera module of GRAMME is most similar to Monodepth2, we provide the performance evaluation for GRAMME models trained using range sensors (for example, lidar and radar), emphasizing the effectiveness of the multimodal approach. Note that none of the models has access to additional sensor measurements at test time other than camera images. The results indicate that GRAMME models distinctly and consistently outperform the other approaches thanks to the fusion model design, and reiterate the robustness of GRAMME to the lack of modalities. The results indicate that exploiting the cross-modal relations is crucial for robust all-weather ego-motion estimation for AVs.

#### Ablation study on the deep network

Deep learning models might benefit from larger and more complex networks to improve the prediction accuracy^41^, which comes at a run-time cost. The encoders in GRAMME are based on ResNet18^42^ architecture. We replace the encoder with commonly used networks such as MobileNet^43^ and VGG16 to analyse the performance and latency of the models^44^, which is shown in Extended Data Fig. 1b. We benchmark the models in terms of depth prediction performance and inference time for a minibatch size of four on an NVIDIA GTX 1080Ti consumer-grade GPU. The inference time is evaluated for the total of pose and depth predictions with an additional pose fusion for the multimodal tests. While the networks have the same inference time for different test conditions, the networks in fusion models have higher latency than the models for single-modality. The multimodal input and parallel network branches for multiple modalities cause a higher latency in fusion models. Although overall run-time for ResNet is higher than MobileNet, ResNet achieves a significant performance boost. On the other hand, despite the slight performance gain of VGG in the monocular setting at the cost of four-times the inference time of ResNet, VGG falls behind ResNet in the other test settings. ResNet efficiently trades off between accuracy and latency, and has a noticeably lower GPU run-time. We, therefore, select ResNet as our encoder.

### Interpretability

We visualize the feature space of the depth prediction module with respect to the camera, lidar, and radar inputs to better understand the multimodal aspect of GRAMME. Figure 4a compares the SHAP values of multimodal depth predictions under different test conditions, and explains the output of GRAMME trained on the Robotcar dataset. While the pixels marked with red points increase the prediction accuracy, blue points decrease it. The input RGB images are shown on the left, and we also place the transparent grey-scale versions of them in the background of each explanation. The sum of the SHAP values for each explanation equals the difference between the current model output and the expected model output that is averaged over the background dataset. Note that the red points for camera predictions are highly scattered, and the blue points are usually concentrated around the occluded and the glaring regions. However, when the same DepthNet model is trained using multiple modalities that are more immune to adverse conditions, the red points are focused more on geometrically meaningful and semantically consistent regions. For example, lidar and radar fusion enables the model features to capture road boundaries, traffic signs, and static objects. Another notable difference is that although the road markings are not clearly visible in the snow, the range sensors attract the model focus to road boundaries. On the other hand, the lidar-based model suffers from the water droplets in fog, visualized by the dense blue points around it. The results validate the effectiveness of GRAMME in exploiting the cross-domain complementary features.

#### Visualizing feature space with SHAP values

SHAP^20^ approximates an interpretable, explanation model *g* of the original, complex model *f*, to explain a prediction made by the model *f*(*x*). SHAP provides post-hoc model explanations for an individual output of *f* and is model-agnostic. SHAP is a game-theoretic approach based on Shapley values^45^, which calculates the contribution of each feature in the final prediction performance. We use a special implementation of the SHAP approach, Deep SHAP method introduced by Lundberg and colleagues^20^, which combines SHAP values computed for smaller components of the network into SHAP values for the whole network. It defines DeepLIFT’s multipliers^46^ in terms of SHAP values, and recursively passes the values backwards through the network. Deep SHAP exploits the composition rule and the efficient analytical SHAP solutions for simple networks components such as linear, max pooling, or an activation function with just one input, enabling a fast approximation of values for the whole model. This approach helps us derive an effective linearisation from the SHAP values computed for each component instead of heuristically choosing ways to linearize components.

### Computational hardware and software

We stored the raw dataset files on multiple hard drives. We performed the demosaicing of camera images, the projection of lidar frames, and Cartesian conversion of radar measurements on Intel Xeon CPUs, which are then stored on a fast local SSD. We used two local NVIDIA RTX 3090 GPUs for each training experiment accelerated through batch parallelization and a local NVIDIA GTX 1080Ti GPU to evaluate run-time performance. We implement our multimodal processing pipeline in Python and employ imaging processing libraries such as colour-demosaicing (v.0.1.6), and pillow (v.8.4.0). To train the deep learning models, and augment the datasets, we used machine learning libraries such as PyTorch (version 1.8.0), torchvision (v.0.9.1). We generated all plots using matplotlib (v3.5.0) and seaborn (v.0.11.2). The Robotcar dataset is processed using Robotcar dataset SDK (v.3.1), and the RADIATE dataset is processed with RADIATE dataset SDK (commit dca2270).

### Supplementary information

Supplementary Information Supplementary Notes 1–6.

## Figures and Tables

**Extended Data Table 1:**
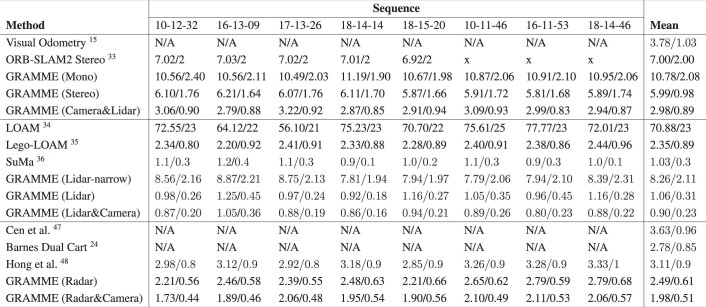
Comparative evaluation with different sensor modalities. Results on the Oxford Radar RobotCar dataset^15^ are given in tuples of (% translation error) / (deg/100m). The column ‘Mean’ shows the mean spatial cross-validation error over the test sequences, ensuring that test and training data are not correlated. Failed sequences are marked with ‘x’. Although GRAMME models with individual modalities slightly outperform the baselines in most sequences, the fusion models significantly improve upon the most related state-of-the-art due to the effective use of additional data

**Extended Data Table 2:**
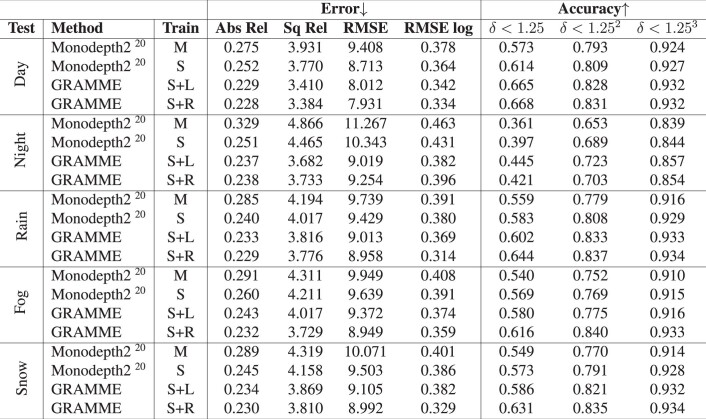
Quantitative comparison with state-of-the-art methods in terms of depth prediction error and accuracy. A higher value is better for the accuracy columns, and a lower value is better for the others. The methods are trained on the daytime data of the Oxford Robotcar dataset^15^ and directly tested under the weather conditions labelled in the test column. The modalities used to train the models are represented by: Monocular (M), stereo (S), lidar (L), and radar (R). The models are tested with monocular images only without access to any additional sensor. Notably, the fusion GRAMME models significantly improve the generalization performance against adverse conditions compared to Monodepth2 that is the most similar architecture to the camera-only GRAMME model

## Data Availability

The Oxford Robotcar Dataset^16^ and the Oxford Robotcar Radar^15^ datasets are available from the University of Oxford under a Creative Commons Attribution-NonCommercial-ShareAlike 4.0 International License (https://robotcar-dataset.robots.ox.ac.uk/). The RADIATE dataset^17^ is available from the Edinburgh Centre for Robotics, Heriot-Watt University, under a Creative Commons Attribution-NonCommercial-ShareAlike 4.0 International License (http://pro.hw.ac.uk/radiate/). The references involve the minimum datasets that are necessary to interpret, verify and extend the research in the article, transparent to readers.

## Code availability

All code was implemented in Python using the deep learning framework PyTorch. Code, trained models and scripts reproducing the experiments of this paper are available at https://github.com/yasinalm/gramme (refs. ^47–49^). All source code is provided under the MIT license.
