# Supplementary material for: Deep learning-based robust positioning for all-weather autonomous driving
Source: Nat Mach Intell. 2022 Sep 8;4(9):749–60. doi: 10.1038/s42256-022-00520-5 (PMC10543073; doi:10.1038/s42256-022-00520-5)
Supplement: Supplementary file 1 — Supplementary Notes 1–6. [file 42256_2022_520_MOESM1_ESM.pdf]

nature portfolio

https://doi.org/10.1038/s42256-022-00520-5

Supplementary information

Deep learning-based robust positioning for all-weather autonomous driving

In the format provided by the authors and unedited

Supplementary Information for

Deep learning-based robust positioning for all-weather autonomous driving

Yasin Almalioglu, Mehmet Turan, Niki Trigoni, and Andrew Markham

Corresponding Author: Yasin Almalioglu.
E-mail: yasin.almalioglu@cs.ox.ac.uk

This PDF file includes:

Supplementary Notes 1-6
SI References

Supplementary Notes

1. Supplementary Note 1: Levels in autonomous driving

There are five safety levels in automotive software to define autonomous driving. The SAE J3016 standard (1) grades vehicle automation using a scale from 0 to 5, which is officially adopted by the U.S. Department of Transportation (2). Lower levels feature essential driver assistance, while higher levels move towards vehicles requiring no human interaction. Level 5 represents a fully automated vehicle capable of performing all driving functions under any conditions.

Supplementary Note 2: The effect of precipitation on lidars

Precipitation reduces free space transmissivity, resulting in distortions on lidar measurements. Laser rays reflected by distant objects are attenuated and may not be received by the sensor, leading to a reduced visible range. Also, dense water droplets back-scatter laser beams and cause scattered measurements. These adverse conditions can cause false point detections and inaccurate distance measurements (3).

Supplementary Note 3: Challenges in deploying radars on AVs

However, there are several challenges in implementing radars that have critical impact the radar performance such as field-of-view (the proportion of the scene visible through radar), angular resolution (the smallest angle between uniquely distinguishable objects) and effective range (the longest detectable distance of an object). For example, radar has intrinsically lower spatial resolution than lidar due to the longer signal wavelength and wide beamwidth, which has limited the use of radars primarily to Doppler-based speed measurements. In recent years, mmWave imaging radars have emerged, enabling measured point clouds to be at a comparable resolution and density as a low-grade lidar. Figure 1 in the main text shows example measurements, where the prominent intensity peaks correspond to objects on the road (e.g., vehicles, walls, trees, and pedestrians). However, radars in the existing autonomous driving datasets are still under-explored compared to cameras and lidars, mainly due to their significant data sparsity issue. For example, each lidar frame in the nuScenes dataset (4) has about 35K points, but each radar frame has only 200 points on average. Similarly, a typical single-chip mmWave radar point cloud can have 100x fewer points than a corresponding lidar scan due to the hardware constraints on the number of antennas (5). Moreover, unlike lidars, the commercial imaging radars deployed on AVs lack the elevation information and measure only range, azimuth and Doppler dimensions, leading to 2D geometric representation of the environment.

Most of the radars used in the existing datasets use conventional electronically steerable antenna arrays, which tend to generate beam patterns with a wide beamwidth ($3.2° - 12.3°$). On the other hand, the DENSE (6) dataset contains a proprietary radar mounted on the front bumper of the vehicle, which has only a 35° angular field of view. Fortunately, recent datasets such as Oxford Radar Robotcar (7) (ORR) and the RADIATE (8) datasets deploy a radar with a mechanically rotating horn antenna, which has high directionality, a much finer spatial resolution of 0.9°, and 360° field of view. The mmWave radar generates dense intensity maps, as shown in Fig. 1, where each pixel represents the reflected signal strength.

Integrating radars into an odometry system to improve reliability requires addressing multiple challenges. Current implementations of FMCW radar sensors suffer from multiple sources of noises such as clutter, sidelobes, multi-path reflections, and receiver saturation since the sensor is susceptible to the surface reflectance and the reflector pose. Multi-path reflection can cause inconsistent measurements between consecutive frames, resulting in additional noise and outliers. Consequently, radar readings tend to be noisier than the camera and lidar data, introducing distinct challenges for ego-motion estimation. In addition, due to the lower angular resolution of radars, adjacent objects might be detected as a single point, which causes a sparser representation of the scene. Such challenges cause failures for conventional methods designed for lidar data (e.g., ICP (9)) when directly applied to mmWave radar data. GRAMME predicts masks to handle the distortions on lidar measurements caused by precipitation, and the inherent noise and the multi-path effect on radar measurements.

Supplementary Note 4: Supervised and self-supervised learning-based approaches

Supervised learning approaches exploit the existence of ground truth data to learn the geometry of the scene from the input. Although supervised-learning approaches (10–12) show high-quality motion and depth estimation results, the acquisition of large-scale ground truth can be imprecise, impractical or even impossible in diverse real-world scenes. Moreover, the loose time synchronisation between the ground truth information and the multi-sensory input data imposes further challenges for the supervised methods (13). In recent years, self-supervised deep learning approaches have achieved remarkable results (14–18), comparable to those from supervised techniques. However, these self-supervised studies have been mostly limited to a single modality, which has severely limited the functionality and robustness under adverse weather conditions. Self-supervised learning approaches are based on the principles of structure from motion (SfM): When the same scene is observed from two different positions, the geometry of the scene will be consistent if a correct depth is assigned to each pixel, and the camera motion (ego-motion) is correctly estimated. However, these methods tend to suffer from the challenges of adverse weather, such as textureless areas, occlusions, and reflections, which may cause too many unknowns for epipolar geometry constraints to disambiguate. In addition, complete view consistency can only be achieved if the discrepancies between the measurements are correctly accounted for. Thus, self-supervised methods based on visible spectrum sensors often rely on additional information.

Supplementary Note 5: Example sensor types supported by GRAMME

For example, in addition to dense lidars (e.g., Velodyne HDL-32E 3D LIDAR 360° HFoV), GRAMME efficiently processes the low-cost alternatives that are mainly designed for obstacle detection (e.g., SICK LD-MRS 3D LIDAR 85° HFoV). Besides, the camera modules can work for both monocular and stereo images from different cameras without any additional configuration for evaluation.

Supplementary Note 6: Observations on the results obtained from the RADIATE dataset

We use the same multi-modal settings for the RADIATE dataset that are used for ORR dataset evaluation. The results on the RADIATE dataset are aligned with our observations on the ORR dataset except with notable differences in rain and snow test conditions. Unlike the ORR dataset, the sequences in both test conditions of the RADIATE dataset might be significantly occluded as the dataset is collected under heavy precipitation, resulting in ultra-low visibility for the camera. In those conditions, the lidar measurements are widely scattered in part, which reduces the performance of the lidar and camera-based model.

References

1. Committee, S. O.-R. A. V. S. *et al.* Taxonomy and definitions for terms related to on-road motor vehicle automated driving systems. *SAE Standard J* **3016**, 1–16 (2014).
2. Automated Vehicles Comprehensive Plan | US Department of Transportation. https://www.transportation.gov/av/avcp.
3. Bijelic, M., Gruber, T. & Ritter, W. A Benchmark for Lidar Sensors in Fog: Is Detection Breaking Down? In *2018 IEEE Intelligent Vehicles Symposium (IV)*, 760–767 (2018).
4. Caesar, H. *et al.* nuScenes: A Multimodal Dataset for Autonomous Driving. In *Proceedings of the IEEE/CVF Conference on Computer Vision and Pattern Recognition*, 11621–11631 (2020).
5. Almalioglu, Y., Turan, M., Lu, C. X., Trigoni, N. & Markham, A. Milli-RIO: Ego-Motion Estimation With Low-Cost Millimetre-Wave Radar. *IEEE Sensors Journal* **21**, 3314–3323 (2021).
6. Bijelic, M. *et al.* Seeing Through Fog Without Seeing Fog: Deep Multimodal Sensor Fusion in Unseen Adverse Weather. In *Proceedings of the IEEE/CVF Conference on Computer Vision and Pattern Recognition*, 11682–11692 (2020).
7. Barnes, D., Gadd, M., Murcutt, P., Newman, P. & Posner, I. The Oxford Radar RobotCar Dataset: A Radar Extension to the Oxford RobotCar Dataset. In *2020 IEEE International Conference on Robotics and Automation (ICRA)*, 6433–6438 (2020).
8. Sheeny, M. *et al.* RADIATE: A Radar Dataset for Automotive Perception in Bad Weather. In *2021 IEEE International Conference on Robotics and Automation (ICRA)*, 1–7 (2021).
9. Chen, Y. & Medioni, G. Object modelling by registration of multiple range images. *Image and Vision Computing* **10**, 145–155 (1992).
10. Eigen, D., Puhrsch, C. & Fergus, R. Depth Map Prediction from a Single Image using a Multi-Scale Deep Network. *Advances in Neural Information Processing Systems* **27**, 2366–2374 (2014).
11. Laina, I., Rupprecht, C., Belagiannis, V., Tombari, F. & Navab, N. Deeper Depth Prediction with Fully Convolutional Residual Networks. In *2016 Fourth International Conference on 3D Vision (3DV)*, 239–248 (IEEE, 2016).
12. Kendall, A., Grimes, M. & Cipolla, R. PoseNet: A Convolutional Network for Real-Time 6-DOF Camera Relocalization. In *Proceedings of the IEEE International Conference on Computer Vision*, 2938–2946 (2015).
13. Praveen Kumar, D., Amgoth, T. & Annavarapu, C. S. R. Machine learning algorithms for wireless sensor networks: A survey. *Information Fusion* **49**, 1–25 (2019).
14. Zhou, T., Brown, M., Snavely, N. & Lowe, D. G. Unsupervised Learning of Depth and Ego-Motion from Video. In *2017 IEEE Conference on Computer Vision and Pattern Recognition (CVPR)*, 6, 6612–6619 (2017).
15. Godard, C., Aodha, O. M., Firman, M. & Brostow, G. Digging Into Self-Supervised Monocular Depth Estimation. In *2019 IEEE/CVF International Conference on Computer Vision (ICCV)*, 3827–3837 (2019).
16. Mahjourian, R., Wicke, M. & Angelova, A. Unsupervised Learning of Depth and Ego-Motion from Monocular Video Using 3D Geometric Constraints. In *2018 IEEE/CVF Conference on Computer Vision and Pattern Recognition*, 5667–5675 (2018).
17. Almalioglu, Y., Saputra, M. R. U., d Gusmão, P. P. B., Markham, A. & Trigoni, N. GANVO: Unsupervised Deep Monocular Visual Odometry and Depth Estimation with Generative Adversarial Networks. In *2019 International Conference on Robotics and Automation (ICRA)*, 5474–5480 (2019).
18. Ozyoruk, K. B. *et al.* EndoSLAM dataset and an unsupervised monocular visual odometry and depth estimation approach for endoscopic videos. *Medical Image Analysis* **71**, 102058 (2021).
